# Beyond Tumor Control: Radiation‐Induced Ascites as a Rare Complication in Gastric Cancer Management

**DOI:** 10.1155/crom/2063269

**Published:** 2026-06-26

**Authors:** Vahid Mohammadkarimi, Abolfazl Khalafi-Nezhad, Nazanin Alemzadeh, Sheyda Sheykhi, Zahra Ghanbarinasab

**Affiliations:** ^1^ Department of Hematology, Medical Oncology and Stem Cell Transplantation, Hematology Research Center, Shiraz University of Medical Sciences, Shiraz, Fars, Iran, sums.ac.ir; ^2^ Digestive Oncology Research Center, Digestive Disease Research Institute, Tehran University of Medical Sciences, Tehran, Tehran Province, Iran, tums.ac.ir

**Keywords:** gastric cancer, nonchylous ascites, radiation-induced injury

## Abstract

**Background:**

Gastric cancer is a leading cause of cancer‐related mortality worldwide, with gastric adenocarcinoma as the predominant subtype. Multimodal treatment includes surgery, chemotherapy, and radiotherapy, which may rarely lead to complications such as ascites.

**Case Presentation:**

A 51‐year‐old male with poorly differentiated gastric adenocarcinoma of the pyloric region underwent chemotherapy and curative resection, achieving a complete pathological response. He subsequently received adjuvant radiotherapy (45 Gy in 25 fractions) and developed acute‐onset ascites. Paracentesis yielded nonchylous fluid with elevated protein, lymphocytic predominance, and negative cytology. In the absence of validated thresholds for radiation‐induced ascites, differentiation relied on excluding common causes using established parameters: serum–ascites albumin gradient (SAAG), polymorphonuclear (PMN) cell count, and adjunctive markers (e.g., ADA, triglycerides, amylase, and BNP) to rule out portal hypertensive, infectious, tuberculous, chylous, pancreatic, and cardiac etiologies. Comprehensive evaluation including PET imaging, repeat laparoscopy with peritoneal biopsies, and follow‐up imaging revealed no recurrence or surgical complications. The ascites resolved after a single paracentesis without recurrence.

**Discussion:**

The diagnosis was made by exclusion, as the case is very rare and no specific biomarkers exist. Proposed mechanisms include endothelial injury, increased vascular permeability, and microthrombotic changes; however, evidence remains inferential.

**Conclusion:**

This case underscores the importance of maintaining clinical vigilance for radiation‐induced ascites in postradiotherapy patients and highlights the need for prospective studies to identify predictive biomarkers and optimize radiation field planning to minimize this rare but clinically significant complication.

## 1. Introduction

Gastric cancer remains a major global health challenge and is currently the fourth leading cause of cancer‐related mortality worldwide, following lung, colorectal, and liver cancers. The vast majority of cases are histologically classified as gastric adenocarcinoma, which accounts for more than 90% of all gastric malignancies and originates from the mucus‐secreting epithelial cells of the gastric mucosa. In Iran, gastric cancer represents one of the most prevalent malignancies and constitutes a significant public health concern. According to GLOBOCAN 2020 data, the age‐standardized incidence rate is approximately 22.4 per 100,000 in men and 12.5 per 100,000 in women, with markedly higher rates reported in certain high‐risk regions—particularly in northern provinces—where incidence may exceed 35 per 100,000, highlighting substantial geographic variability within the country [1, 2]. This tumor typically develops insidiously, with early stage disease often presenting with vague and nonspecific symptoms, leading to delayed diagnosis. Common clinical manifestations include unintentional weight loss, epigastric pain, nausea, vomiting, anorexia, and gastrointestinal bleeding such as hematemesis or melena; in advanced stages, patients may also develop dysphagia or odynophagia [3].

The therapeutic strategy for gastric cancer is largely determined by the tumor′s anatomical location, histopathological characteristics, and stage at diagnosis. Management typically involves a multimodal approach, including surgical resection, systemic chemotherapy, radiation therapy, targeted molecular agents, immunotherapy, and supportive or palliative care [4].

Radiation therapy, while beneficial in both curative and palliative settings, is associated with a spectrum of adverse effects. These are generally classified as either acute or chronic, depending on the timing of onset relative to treatment. The most commonly reported complications of abdominal or pelvic radiotherapy include gastrointestinal symptoms such as diarrhea, nausea, and weight loss, along with urinary disturbances and abdominal or pelvic pain. Reproductive and sexual dysfunction may also occur, particularly in patients receiving pelvic irradiation. In addition to these local effects, systemic manifestations such as radiation‐induced dermatitis and fatigue are frequently observed and can substantially impair overall quality of life [5].

An infrequent but clinically relevant complication of abdominal radiotherapy is ascites, a condition characterized by the accumulation of fluid within the peritoneal cavity. Though rare, this entity has been documented in several case reports, often in the context of malignancy‐associated lymphatic disruption [6, 7].

The current report presents a unique case of nonchylous ascites secondary to radiotherapy in a male patient with gastric adenocarcinoma, highlighting the need for heightened clinical awareness of this rare but potentially serious complication.

## 2. Case Presentation

### 2.1. Patient Information

A 51‐year‐old Iranian male was admitted to the Hematology and Oncology Department of Fars University of Medical Sciences in Shiraz, Iran, following referral to a gastroenterology specialist for evaluation of unexplained and progressive weight loss. He presented with associated symptoms of nausea, vomiting, and anorexia, while denying epigastric pain, hematemesis, or melena.

The patient had no known comorbidities and reported no history of smoking, alcohol consumption, or regular medication use. His family history was negative for malignancy and other relevant diseases.

### 2.2. Clinical Findings and Diagnosis

On October 14, 2023, upper gastrointestinal endoscopy and colonoscopy were performed. Endoscopic findings revealed extensive ulceration and luminal narrowing of the pyloric channel and distal antrum, raising strong suspicion for malignancy.

**Figure 1:**
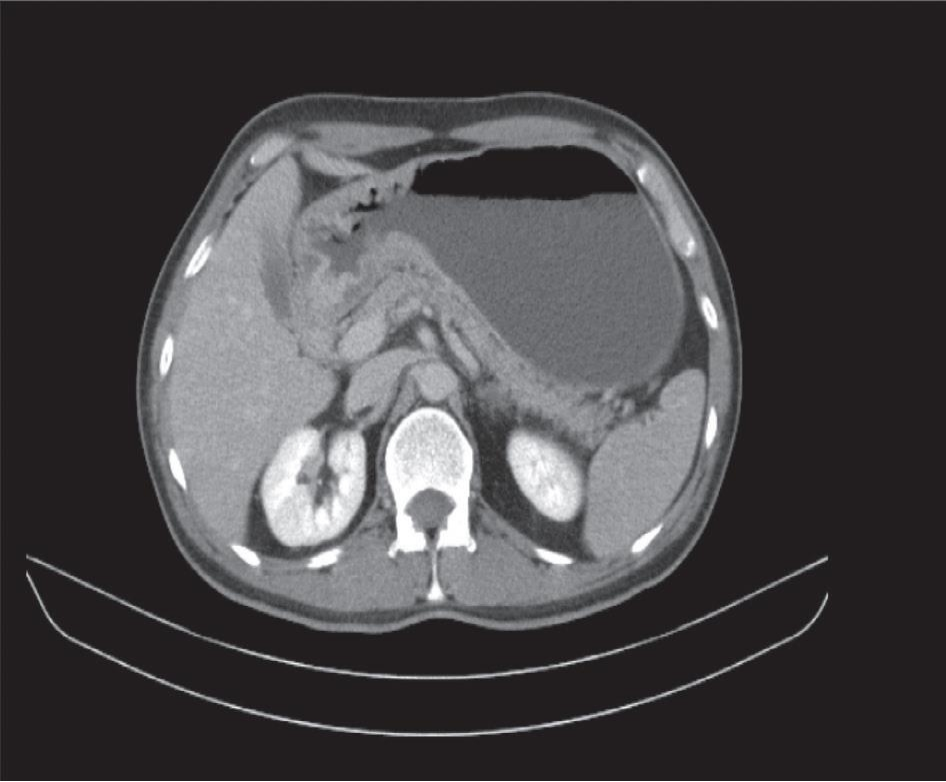
Diagnostic CT showing diffuse thickening of the duodenal and jejunal walls.

To further characterize the lesion, a contrast‐enhanced computed tomography (CT) scan was obtained, and endoscopic biopsy specimens were submitted for histopathological analysis. The CT scan demonstrated marked and diffuse thickening of the duodenal and jejunal walls and a perigastric lymph node measuring 8 × 5 mm in diameter (Figure 1). Histopathological evaluation confirmed the presence of poorly differentiated adenocarcinoma, establishing a diagnosis of gastric cancer.

For staging purposes, a diagnostic laparoscopy was performed on October 19, 2023. The procedure showed no evidence of peritoneal carcinomatosis, ascites, pancreatic adhesions, or distant metastases. The tumor was localized to the pyloric region, resulting in partial gastric outlet obstruction. The patient was staged as T3N1M0, corresponding to Stage IIIA disease.

### 2.3. Therapeutic Intervention

The patient subsequently received eight cycles of chemotherapy with the FLOT regimen (5‐fluorouracil, leucovorin, oxaliplatin, and docetaxel), which were completed by March 2, 2024. After four cycles of neoadjuvant chemotherapy, on December 27, 2023, he underwent surgical resection. The patient underwent subtotal gastrectomy with Roux‐en‐Y gastrojejunostomy and jejunojejunostomy reconstruction, along with regional lymphadenectomy (six lymph nodes dissected) and resection of approximately 2 cm of the proximal duodenum distal to the pylorus. The resected specimen was submitted for histopathological analysis, which revealed a complete pathological response, characterized by the absence of residual tumor, negative resection margins, and no viable malignant cells in the stomach. Additionally, all examined lymph nodes were free of metastatic involvement.

### 2.4. Adverse Event and Management

**Figure 2:**
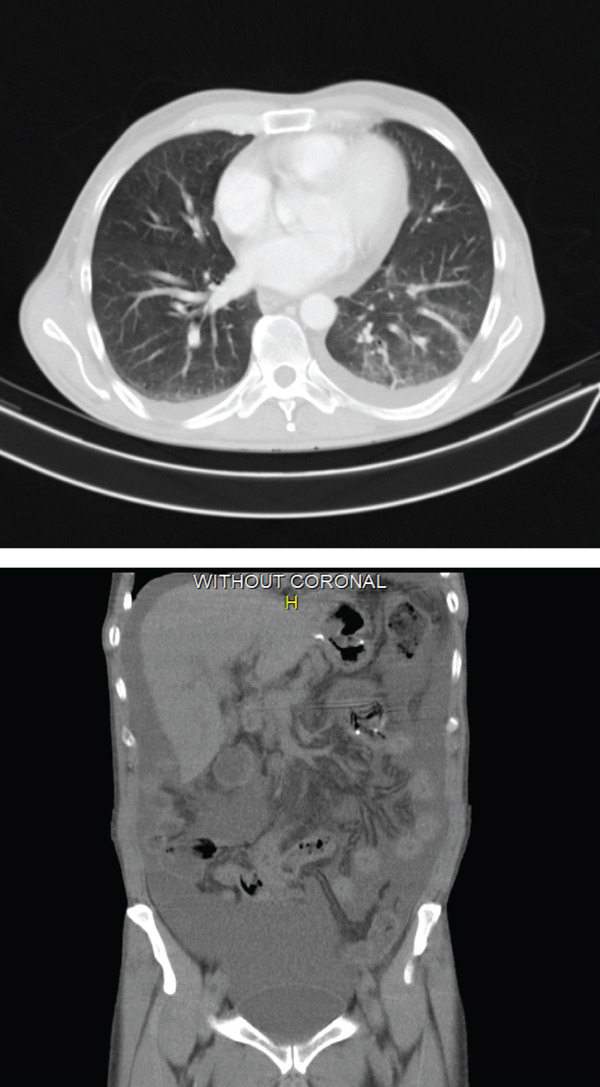
CT during ascites showing pleural effusion and free fluid.

Between March 26 and April 30, 2024, the patient underwent 25 sessions of adjuvant radiotherapy. Radiotherapy was delivered using a three‐dimensional conformal radiotherapy (3D‐CRT) technique. The prescribed treatment volume received a total dose of 45 Gy in 25 fractions, corresponding to 1.8 Gy per fraction. Approximately 1 week after completing radiotherapy, he developed acute‐onset ascites without an identifiable cause. Ultrasonography revealed a large volume of free intraperitoneal fluid in the abdominopelvic cavity. Also, CT showed minimal pleural effusion on the right side and minimal to mild pleural effusion on the left side associated with adjacent lung collapse. Moderate to severe free ascitic fluid was seen in the abdomen and pelvic cavity (Figure 2).

Diagnostic paracentesis was performed, yielding 2100 mL of yellow‐tinged ascitic fluid. Cytopathological analysis of the fluid showed the following results (Table 1).

**Table 1:** Laboratory findings of ascitic fluid and corresponding serum values.

Parameter	Ascitic fluid	Serum	Interpretation/notes
**Glucose (mg/dL)**	147 ↑	—	Elevated compared to typical ascites values
**Protein (g/dL)**	3.72	—	Moderately high
**Total cell count (/*μ*L)**	750	—	Mildly elevated
**WBC (/*μ*L)**	160 (> 95% lymphocytes)	—	Lymphocyte predominance
**LDH (U/L)**	182.6	—	Within a moderate range
**Albumin (g/dL)**	2.37	4.0	Lower in ascitic fluid
**Globulin (g/dL)**	—	3.0	Normal
**A/G ratio**	—	1.33	Normal

Following this episode, the patient experienced no recurrence of ascites. Given the atypical presentation, additional investigations were conducted to find out the cause and rule out disease recurrence or peritoneal metastasis. The ascitic fluid profile in this patient demonstrated a high serum–ascites albumin gradient (SAAG) of 1.63 g/dL in conjunction with an elevated protein concentration (3.72 g/dL), a pattern classically associated with cardiac etiologies and hepatic venous outflow obstruction rather than portal hypertension alone. Congestive heart failure and cardiac dysfunction were excluded by echocardiography, which confirmed normal cardiac structure and function. Constrictive pericarditis was considered and excluded based on the absence of pericardial calcification on both echocardiography and contrast‐enhanced CT, with no hemodynamic features suggestive of constrictive physiology. Budd–Chiari syndrome and IVC obstruction were excluded through serial contrast‐enhanced CT imaging performed throughout the patient′s oncological follow‐up, which demonstrated patent hepatic veins and IVC without thrombosis or extrinsic compression. Additionally, the elevated ascitic glucose level, in the absence of diabetes mellitus, was interpreted as reflecting local metabolic dysregulation or altered membrane transport secondary to radiation‐induced tissue injury, rather than an infectious or malignant process.

Whole‐body positron emission tomography (PET) demonstrated no evidence of metabolically active malignancy.

**Figure 3:**
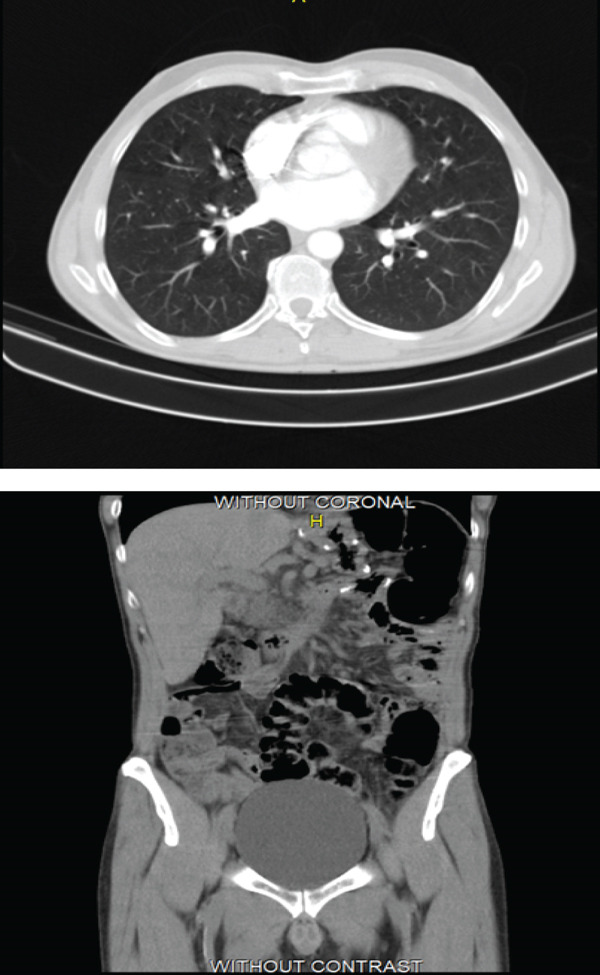
CT after abdominal TAP showing no abnormal radiological findings.

For further evaluation, a second diagnostic laparoscopy was performed on October 5, 2024. Peritoneal biopsies obtained during the procedure were negative for malignancy on histopathological examination. Additionally, a follow‐up CT scan on January 11, 2025, showed clear lung fields with no pleural effusion and no discrete mass (Figure 3).

### 2.5. Follow‐Up and Outcome

Based on a comprehensive multidisciplinary review and after systematically excluding all other potential etiologies through thorough clinical, laboratory, and imaging evaluations, the ascites was attributed to postradiotherapy inflammatory changes rather than tumor recurrence or metastatic disease. The patient was followed systematically after the resolution of ascites. Follow‐up was conducted every 2 months during the first 6 months, every 3 months during the subsequent 6 months, and every 4 months thereafter.

At present, with a total follow‐up duration of approximately 19 months, the patient has shown no recurrence of ascites and remains cancer‐free. This duration of follow‐up provides reassuring evidence of sustained resolution; however, longer follow‐up is generally advisable to definitively exclude late recurrence, particularly in oncologic settings (Table 2).

**Table 2:** Timeline of diagnosis and treatment interventions.

Event	Date	Details
Initial presentation	Early October 2023	Progressive weight loss, gastroenterology referral
Endoscopy and biopsy	October 14, 2023	Ulcerative lesion, suspicion of malignancy
Diagnosis confirmed	October 2023	Poorly differentiated gastric adenocarcinoma
Staging laparoscopy	October 19, 2023	No metastasis, no carcinomatosis
Start of chemotherapy	October 2023	Neoadjuvant chemotherapy initiated
Surgery (gastrectomy)	December 27, 2023	After four cycles, complete response
Completion of chemotherapy	March 2, 2024	Eight cycles completed
Adjuvant radiotherapy	March 26–April 30, 2024	25 sessions, 45 Gy total
Onset of ascites	Early May 2024	Acute‐onset postradiotherapy
Paracentesis	May 2024	2100 mL fluid drained
PET scan	2024	No active malignancy
Second laparoscopy	October 5, 2024	No malignancy found
Follow‐up CT scan	January 11, 2025	No effusion, no recurrence
Current status	Latest follow‐up	No recurrence, stable condition

## 3. Discussion

This case report concerns a 51‐year‐old male diagnosed with gastric adenocarcinoma. He underwent chemotherapy, radiotherapy, and laparoscopic surgery as part of his treatment regimen. After completing radiotherapy, the patient presented with unexpected ascites. A diagnostic and therapeutic paracentesis was performed. To further investigate, a PET scan and diagnostic laparoscopy were conducted, both of which showed no signs of malignancy. Notably, the ascites did not recur thereafter. Based on these findings and ruling out other probable causes, we hypothesize that the ascites was most likely attributable to radiotherapy.

A review of the literature reveals few similar cases. In 2009, a 64‐year‐old male with unresectable liposarcoma developed ascites after undergoing radiotherapy, which was resolved with the placement of a shunt. Additionally, between 1981 and 1987, 207 patients who received whole‐abdominal radiotherapy for gynecologic malignancies were studied. Of these, 3% developed malignancy‐associated ascites within 12 months of treatment. Most cases were managed with observation and diuretics, though two patients required multiple paracentesis procedures. Barrera et al. reported a similar case of recurrent ascites following adjuvant abdominal radiotherapy in a patient with gastric adenocarcinoma, where malignancy and infection were excluded. However, their case was confirmed as *chylous* ascites caused by disruption of the cisterna chyli—a mechanistically distinct entity from the nonchylous, lymphocyte‐predominant ascites observed in our patient, which points toward radiation‐induced endothelial injury rather than lymphatic disruption. To our knowledge, the present case represents the first reported nonchylous radiation‐induced ascites in gastric adenocarcinoma following 3D‐CRT [7–9].

In this case, several differential diagnoses were considered to explain the patient′s ascites.

The first possibility was metastasis. This diagnosis was ruled out following a thorough diagnostic workup, including a PET scan, CT scan, and diagnostic laparoscopy, all of which showed no evidence of metastatic disease. These findings effectively excluded the possibility of ascites being caused by peritoneal metastasis.

The second potential cause was postsurgical complications. Since the ascites did not develop immediately after surgery, this raised doubts about a direct postoperative etiology. Postoperative complications following gastrectomy predominantly occur within the early postoperative period, typically within 30–90 days, with large cohort data demonstrating that the majority of morbidity arises during this timeframe. In contrast, the development of clinically significant ascites more than 6 months after surgery is exceedingly rare, and reliable data on its incidence are lacking. Although minimal pelvic free fluid may occasionally be observed on follow‐up imaging, it is generally considered clinically insignificant and should not be interpreted as true postoperative ascites [10, 11].

Moreover, a diagnostic laparoscopy was performed, which ruled out any vascular injury or significant anatomical disruption as a consequence of the previous surgery. Given the temporal separation between the surgery and the onset of ascites, along with the absence of vascular injury on laparoscopy, postsurgical complications were deemed unlikely. Finally, if ascites had been due to anatomical injury or lymphatic disruption, we would have expected the condition to recur after a single TAP procedure. However, the patient experienced resolution of ascites following the initial TAP, without recurrence. This finding further supports the idea that ascites was not due to persistent anatomical injury, as typically, such injuries would result in recurrent fluid accumulation after a single drainage.

Taken together, these comprehensive diagnostic investigations effectively ruled out metastasis, postsurgical complications, and anatomical injury, leading us to conclude that the ascites was most likely a radiotherapy‐induced complication.

The resolution of ascites following a single paracentesis and cessation of radiotherapy suggests that removal of the inciting stimulus—rather than the drainage procedure itself—was the primary driver of recovery. Paracentesis therefore likely served a supportive role, relieving symptomatic fluid burden without directly addressing the underlying pathophysiology. Fluid reabsorption appears to have been predominantly passive, occurring as radiation‐induced endothelial injury subsided over time, rather than reflecting any measurable therapeutic modulation of the inflammatory cascade. This interpretation is supported by serial ESR measurements, which showed only a minimal decline from 15 to 10 mm/h—both values remaining well within the normal range (< 20 mm/h)—indicating the absence of a significant inflammatory shift during the resolution period.

Radiotherapy is known to induce endothelial and microvascular injury, primarily mediated through inflammatory signaling pathways and alterations in the local tissue microenvironment. These changes can lead to endothelial dysfunction, vascular instability, and increased permeability. However, such mechanisms are not disease‐specific and do not establish a direct causal relationship between microvascular injury, microthrombosis, and ascites formation; therefore, they should be considered biologically plausible rather than definitive [12, 13].

At the tissue level, these primary effects translate into inflammatory and microenvironmental alterations. Activation of inflammatory signaling pathways and epithelial–mesenchymal transition (EMT) contributes to chronic inflammation, fibrosis, and progressive microvascular dysfunction. Even at conventional therapeutic doses (e.g., 45 Gy), subclinical endothelial injury and gradual tissue remodeling may occur in the absence of overt structural abnormalities. The extent of injury is further influenced by tissue‐specific radiosensitivity. Tissues with high epithelial turnover and dense microvascular networks are particularly vulnerable to these effects. In this context, the peritoneum and splanchnic vasculature may be especially susceptible, where increased vascular permeability, impaired lymphatic drainage, and persistent low‐grade inflammation can promote fluid accumulation and lead to ascites formation [14].

Several molecular and immunological pathways may further exacerbate this process, but since no direct mechanistic studies exist for radiation‐induced ascites specifically, these pathways are extrapolated from the malignant ascites literature and should be interpreted with caution.

Vascular endothelial growth factor (VEGF) plays a central role in increasing endothelial permeability and facilitating fluid leakage, even in nonmalignant conditions. VEGF promotes endothelial leakage and fluid accumulation, even in tumor‐free environments. Experimental studies have shown that inhibiting VEGF can reduce ascites formation, highlighting its central role in this process [15, 16].

The immune system also plays a crucial role in ascites development. Tumor‐induced immunosuppression and chronic inflammation contribute to the pathological environment. Elevated levels of interleukin‐6 (IL‐6), tumor necrosis factor (TNF), dysfunctional T cells, and M2 macrophages enhance vascular permeability and create a tumor‐supportive microenvironment [17, 18].

Furthermore, additional mechanisms may contribute to fluid retention, such as the activation of the renin–angiotensin–aldosterone system (RAAS), which exacerbates fluid retention [19]. Matrix metalloproteinases (MMPs) also facilitate tumor invasion and vascular leakage. Although promising in preclinical studies, MMP inhibitors are not yet widely used in clinical practice [20].

The proposed mechanisms remain speculative, and further rigorous studies are required to clarify the exact cause of this rare complication.

To prevent the occurrence of radiation‐induced ascites, it is crucial to carefully adjust the radiation dose and optimize the treatment field. One effective strategy is to reduce the radiation dose delivered to normal tissues, particularly in areas surrounding the tumor. By minimizing exposure to nontargeted tissues, the risk of vascular injury and subsequent inflammation that may lead to ascites can be significantly decreased.

Emerging radiotherapy techniques may help mitigate these adverse effects. FLASH radiotherapy (FLASH‐RT), characterized by ultrahigh dose rates, has shown potential in reducing normal tissue toxicity by limiting oxidative stress and inflammatory responses while preserving antitumor efficacy. Although clinical evidence remains limited, this approach may offer a promising strategy for reducing rare complications such as radiation‐induced ascites [14].

Other recommended advanced radiotherapy techniques are intensity‐modulated radiation therapy (IMRT) and stereotactic body radiotherapy (SBRT). These methods allow for more precise targeting of the tumor, ensuring that the radiation is confined to the malignancy while sparing healthy organs and tissues [21, 22]. By carefully delineating the treatment volume and avoiding excessive radiation exposure to the peritoneal cavity and surrounding vessels, the risk of developing portal hypertension and fluid accumulation can be minimized.

## 4. Recommendations for Clinicians Managing Similar Cases

As previously discussed, the hypotheses for radiation‐induced ascites are the development of microinflammation and microthrombosis. Therefore, it is important for clinicians to monitor relevant inflammatory and thrombotic markers throughout the course of treatment, including during radiotherapy.

Although monitoring systemic inflammatory and thrombotic markers during radiotherapy may offer supportive clinical information, their nonspecific nature limits their utility in reliably identifying patients at risk for radiation‐induced complications such as ascites. To better characterize this rare entity, a large prospective cohort study involving patients with gastric cancer undergoing radiotherapy would be of considerable value. Such a study could systematically assess incidence, timing, and potential risk factors through longitudinal follow‐up, integrating clinical evaluation, imaging, and laboratory parameters. This approach may help clarify temporal relationships and provide more robust evidence to distinguish treatment‐related effects from disease recurrence or other confounding conditions, thereby enhancing the current understanding of this complication.

## 5. Conclusion

This case highlights radiation‐induced ascites as a rare but clinically significant complication following adjuvant therapy for gastric adenocarcinoma. Comprehensive diagnostic evaluation effectively excluded recurrent or metastatic disease, suggesting that the ascites was likely related to radiotherapy‐associated microvascular and inflammatory injury, although no direct mechanistic evidence is available. Given the scarcity of reported cases, this presentation underscores the importance of clinical vigilance when new‐onset ascites arises in the postradiotherapy setting. While inflammatory and thrombotic markers were assessed, their nonspecific nature and lack of predictive value in this context limit their utility for early detection or guiding intervention. Further investigation is warranted to elucidate the underlying mechanisms of radiation‐induced ascites and to develop evidence‐based strategies to minimize treatment‐related morbidity while preserving therapeutic efficacy.

## Author Contributions

All authors contributed to the collection of data, manuscript development, and final approval. **Vahid Mohammadkarimi**: methodology (equal), conceptualization (lead), project administration (equal), resources (equal), validation (equal), and supervision (equal); **Abolfazl Khalafi-Nezhad**: methodology (equal), conceptualization (lead), and supervision (equal); **Nazanin Alemzadeh**: writing—original draft (lead), writing—review and editing (lead), and resources (equal); **Sheyda Sheykhi**: data curation (equal), investigation (equal), and writing—original draft (lead); **Zahra Ghanbarinasab**: data curation (equal), investigation (equal), and project administration (equal).

## Funding

No funding was received for this manuscript.

## Ethics Statement

The current study was approved by the Ethics Committee of Shiraz University of Medical Sciences (IR.SUMS.MED.REC.1405.065). Written informed consent was obtained from the patient for participation in this study and for publication of this case report. A copy of the signed consent form is available for review by the journal editor upon request.

## Consent

Written informed consent has been obtained from the patient to publish this paper.

## Conflicts of Interest

The authors declare no conflicts of interest.

## Data Availability

The data are available from the corresponding author upon reasonable request.
